# The effect of deoxynivalenol-contaminated corn and an immune-modulating feed additive on growth performance and immune response of nursery pigs fed corn- and soybean meal-based diets

**DOI:** 10.1093/tas/txab141

**Published:** 2021-09-06

**Authors:** Élise Lafleur Larivière, Cuilan Zhu, Sabrina Zettell, Robert Patterson, Niel A Karrow, Lee-Anne Huber

**Affiliations:** 1 Department of Animal Biosciences, University of Guelph, Guelph, ON N1G 2W1, Canada; 2 Canadian Bio-Systems Inc., Calgary, AB T2C 0J7, Canada

**Keywords:** deoxynivalenol, growth performance, immune response, low-complexity diets, nursery pigs

## Abstract

One hundred eighty newly weaned pigs (21 days of age; 6.9 ± 0.2 kg BW) were used to determine the effects of deoxynivalenol- (DON) contaminated corn and an immune-modulating feed additive on growth performance and immune response of nursery pigs fed corn- and soybean meal-based diets. Pens were randomly assigned to one of five diets: a high-complexity (HC; containing animal protein sources) or one of four low-complexity diets (LC; containing soybean meal as the main protein source) arranged in a 2 × 2 factorial with low (lDON; average 1.4 ppm) or high (hDON; average 3.5 ppm) DON and with or without a feed additive (2 g/kg in complete feed; *n* = 6 pens per treatment) provided in a three-phase feeding program. On day 7, small intestinal histomorphology was assessed in two pigs per pen. On days 8 and 25, two pigs per pen were immunized with ovalbumin (OVA). Blood was collected on days 8, 25, and 38 for determination of OVA-specific IgG. There were no corn type by feed additive interactions or feed additive effects for growth performance. The ADG, ADFI, and G:F in phase I were not different for pigs fed hDON vs. lDON, but were less than those fed the HC diet (contrasts; *P* < 0.05). Over the entire nursery period, ADG and ADFI were less for pigs fed hDON vs. those fed lDON (407 vs. 484 g and 651 vs. 769 g, respectively; *P* < 0.05), ADG was less for pigs fed hDON vs. HC (496 g; *P* < 0.05), and pigs fed lDON had ADG and ADFI not different from those fed the HC diet. Pigs fed hDON had lower final BW than those fed lDON (24.6 vs. 27.6 kg; *P* < 0.01) and tended to have lower final BW than pigs fed the HC diet (27.3 kg; contrast; *P* = 0.052); final BW was not different between pigs fed lDON and HC diets. Jejunal villus heights were shorter for pigs fed hDON and lDON compared to pigs fed HC (438 and 466 vs. 538 µm; contrasts; *P* < 0.05 and *P* = 0.090, respectively) and the villus:crypt ratio tended to be less for pigs fed hDON vs. those fed HC (1.87 vs. 2.22; contrast; *P* = 0.091). On day 38, plasma OVA-specific IgG 1 tended to be less for pigs fed hDON compared to HC (contrast; *P* = 0.075) and OVA-specific total IgG were less for pigs fed LC diets without the feed additive vs. HC (*P* < 0.05). Therefore, high DON (~3.5 ppm) in LC nursery diets interfered with compensatory growth and the humoral immune response. The feed additive did not rescue growth performance, regardless of DON contamination level in LC nursery diets.

## INTRODUCTION

During the early post-weaning period, pigs experience a marked reduction in feed intake and subsequent growth lag, in part due to exposure to food allergens and novel pathogens, an immature gastrointestinal tract, and other environmental stressors (Lallès et al., 2004). Thus, nursery diets typically contain highly digestible and expensive ingredients (e.g., animal protein sources) to combat this reduction in growth performance (Campbell and Dunkin, 1983). Conversely, after an initial reduction in ADG and ADFI, nursery pigs fed simple corn and soybean meal (SBM)-based diets can achieve a BW that is not different from pigs fed nursery diets that contain highly digestible ingredients via compensatory growth (Taylor et al., 2013; Skinner et al., 2014). However, simple plant-based diets may compromise immune function, since plant proteins, including those from SBM, have been linked to transient allergic and inflammatory responses in nursery pigs (Li et al., 1991; Chen et al., 2011). Furthermore, using mainly plant-based ingredients to supply both protein and energy in swine diets results in greater inclusion levels of cereal grains (e.g., corn). These cereal grains can become contaminated with mycotoxins, the secondary metabolites of fungi produced during instances of crop stress (i.e. nutritional deficiency, drought, or excessive humidity; Rodrigues and Naehrer, 2012). One of the most common mycotoxins to infect cereal grains in temperate climates is deoxynivalenol (DON; Halawa et al., 2013; Lessard et al., 2015; Jin et al., 2017). Ingestion of feed contaminated with greater than 1 ppm of DON has been linked to poor growth performance, gut damage, and impaired immune function in pigs (Lessard et al., 2015; Van Le Thanh et al., 2015; Wu et al., 2015), which may render the pigs unable to express compensatory growth. Supplementing simple DON-contaminated nursery diets with immune-modulating and anti-oxidant-containing feed additives may be a strategy to counter the negative effects of DON (Van Le Thanh et al., 2015) and to facilitate compensatory growth, even during instances of DON contamination. Therefore, the aim of the current study was to evaluate the effects of simple plant-based diets, DON contamination, and a feed additive containing immune-modulating components on growth performance, gut morphology, and indices of immune function in nursery pigs.

## MATERIALS AND METHODS

The experimental protocol was approved by the University of Guelph Animal Care Committee (AUP #4404) and followed Canadian Council of Animal Care guidelines (CCAC, 2009). The study was conducted at the Arkell Swine Research Station at the University of Guelph (Guelph, ON, Canada).

### Animals and Experimental Diets

One hundred eighty Yorkshire × Landrace × Duroc pigs (90 castrated males and 90 females) were weaned over two blocks at 21 days of age (6.9 ± 0.2 kg BW). Weaned pigs were divided into 30 pens, with six pigs per pen (three castrated males and three females; balanced for BW and assigning littermates to different pens). Using a randomized complete block design, pens were assigned to one of five dietary treatments (*n* = 6 pens per dietary treatment; study day 0), which were fed over three phases. Phases I, II, and III were fed between study days 0 and 7, 8 and 21, and 22 and 42, respectively. Phase I diets were provided as a crumble and phases II and III diets were pelleted. Pigs had ad libitum access to feed via a four-space feeder and to water via a nipple drinker in each pen. Individual pig BW and per-pen feed disappearance were recorded weekly to determine ADG, ADFI, and G:F in each phase.

A high complexity (HC) nursery diet containing multiple highly digestible protein sources (e.g., whey, fishmeal, and blood products) was used as the control diet (Table 1). The remaining four diets were low complexity (i.e. simple; contained soybean meal as the main protein source and only had low inclusion levels of fishmeal and whey in phase I; LC), and were created according to a 2 × 2 factorial design with DON contamination [low (lDON) and high (hDON)] and the inclusion of a feed additive containing a blend of immune-modulating components [with or without (**+**/−); included in complete feed at 2 g/kg; the feed additive blend contained per kilogram: vitamins (vitamin D_3_: min. 39,650 I.U.; vitamin E: min. 2,600 I.U.; niacin: min. 1,900 mg; thiamine: min. 440 mg; riboflavin: min. 330 mg; calcium d-pantothenate: min. 1,000 mg; pyridoxine: 220 mg; biotin: 1,000 µg; vitamin B_12_: 2,000 µg; menadione: min. 80 mg), yeast product (dehydrated yeast autolysate), and an inorganic adsorbent (montmorillonite clay); NutraMix, Canadian Bio-Systems Inc., Calgary, AB, Canada] as the factors. The LC diets were formulated using corn with low (<1 ppm) and high (>15 ppm) DON contamination. The lDON and HC diets used only corn with low DON contamination. The hDON diets contained a blend of the low and high DON-contaminated corn to achieve the desired DON contents of 3, 4, and 5 ppm in the complete feed for phases I, II, and III, respectively. All other cereal-grain and legume ingredients were also analyzed for mycotoxin contamination and contained minimal amounts of DON (data not shown). Diets were formulated to meet or exceed estimated nutrient requirements for nursery pigs (NRC, 2012; Table 1).

**Table 1.** Ingredient and calculated nutrient composition of experimental diets (as-fed basis)^*a*^

Item	HC			LC^*b*^		
	Phase I	Phase II	Phase III	Phase I	Phase II	Phase III
Ingredient, %						
Corn	16.30	37.90	50.07	46.72	49.47	47.20
Wheat	–	–	–	10.00	10.00	10.00
Barley	25.00	25.00	20.00	–	–	–
Soybean meal, dehulled	13.00	16.00	22.00	24.00	34.00	37.00
Oat groats	10.00	–	–	–	–	–
Fishmeal	5.00	3.00	–	5.00	–	–
Whey, dried	20.00	8.00	–	8.00	–	–
Blood meal	–	2.00	2.00	–	–	–
Blood plasma^*c*^	4.50	2.00	–	–	–	–
Animal-vegetable fat	3.50	3.00	2.50	2.50	2.50	2.50
ʟ -lysine·HCl	0.41	0.37	0.38	0.47	0.35	0.10
dʟ-methionine	0.16	0.16	0.14	0.06	0.11	–
ʟ-tryptophan	–	0.01	0.01	0.02	–	–
ʟ -threonine	0.13	0.13	0.14	0.13	0.09	–
Limestone	0.50	0.58	0.86	1.00	1.18	1.10
Salt	0.10	0.20	0.30	0.20	0.30	0.30
Monocalcium phosphate	0.80	1.05	1.00	1.30	1.40	1.20
Vitamin and mineral Premix^*d*^	0.60	0.60	0.60	0.60	0.60	0.60
Calculated nutrient composition^*e*^						
NE, kcal/kg	2610	2552	2513	2547	2492	2478
Crude protein, %	21.34	20.31	19.23	21.09	21.87	22.75
SID^*f*^ Lys, %	1.46	1.34	1.21	1.40	1.31	1.19
Calcium, %	0.85	0.76	0.69	1.05	0.91	0.85
Total P, %	0.83	0.75	0.63	0.86	0.76	0.73

^*a*^Experimental diets: (1) high complexity (HC) diet containing multiple, highly digestible protein sources, (2) low complexity (LC) diets containing plant proteins as the main sources of protein, without (–) or with (+) the feed additive. Corn with high and low deoxynivalenol (DON) contents was used to create high and low contents of DON in the LC diets to generate four diets according to a 2 × 2 factorial design with the feed additive and DON content as the factors. Phases I, II, and III were fed between study days 0 and 7, 8 and 21, and 22 and 42, respectively.

^*b*^In the LC diets containing the feed additive (+), the feed additive replaced corn (at 0.2%). The feed additive contained a blend of vitamins and adsorbents. (NutraMix; Canadian Bio-Systems Inc., Calgary, AB, Canada).

^*c*^AP920; manufactured by APC Nutrition Inc. (Ames, IA).

^*d*^Provided, per kilogram of diet, 12,000 IU vitamin A as retinyl acetate, 1,200 IU vitamin D3 as cholecalciferol, 48 IU vitamin E as dl-α-tocopherol acetate, 3 mg vitamin K as menadione, 18 mg pantothenic acid, 6 mg riboflavin, 600 mg choline, 2.4 mg folic acid, 30 mg niacin, 18 mg thiamine, 1.8 mg pyridoxine, 0.03 mg vitamin B12, 0.24 mg biotin, 1,200 mg Ca from CaCO_3_, 18 mg Cu from CuSO_4_∙5H_2_O, 120 mg Fe from FeSO4, 24 mg Mn from MnSO_4_, 126 mg Zn from ZnSO_4_, 0.36 mg Se from Na_2_SeO_3_, and 0.6 mg I from KI (DSM Nutritional Products Canada Inc., Ayr, ON, Canada).

^*e*^Calculated using the NRC (2012) model ingredient values.

^*f*^Standardized ileal digestible.

### Assessment of Gut Morphology and Immune Function

On study day 7, 12 pigs per treatment (one castrated male and one female per pen) were randomly selected and euthanized with 3 mL of Euthasol (Virbac, TX) for tissue collection. The entire gastrointestinal tract was immediately removed and gut and organ weights were measured. Five-centimeter segments of the jejunum (1.5 m distal to the ligament of Trietz) and the ileum (0.5 m proximal to the ileo-cecal junction) were rinsed with saline and placed in 10% formalin. The tissue segments were prepared according to Carleton et al. (1980) for histological analysis. Using a Leica DMR fluorescence microscope (Leica Microsystems Inc., Wetzlar, Germany) and Openlab Computer Imaging System (Perkin Elmer, Waltham, MA), villi height and crypt depth were measured for the five longest villi per intestinal section.

On days 8 and 25, an additional 12 pigs per treatment (one castrated male and one female per pen) were randomly selected and vaccinated via intramuscular injection with 0.5 mg ovalbumin (OVA) using 0.5 mg Quil A as the adjuvant in 1 mL of saline (Sigma-Aldrich Co., St. Louis, MO). On day 38, vaccinated pigs were intradermally injected with OVA (100 mg of OVA in 100 µL of saline) on an inner thigh of the hind leg. One hundred microliters of saline was also injected at least five centimeters from the OVA test site to serve as a control. Skinfold thickness measurements were taken immediately before (0 h) and 6-, 24-, and 48-h post-injection using calipers (Model RH15 9LB, Creative Health Products Inc., Ann Arbor, MI). The changes in skinfold thickness (mm; SFT) over time were compared to the saline-injection measurement and were used to determine the dermal hypersensitivity response (DHR; Eq. 1):


$$\begin{array}{l} DHR=\left(SFT_{t_{x}}-SFT_{t_{0}}\right)-(S_{t_{x}}-S_{t_{0}}) \end{array}$$

where SFT_*t*χ_ was the skinfold thickness of the OVA injection site at time *x*, SFT_*t*0_ was the skinfold thickness of the OVA injection site at time 0, S_*t*χ_ was the skinfold thickness of the saline injection site at time *x*, and S_t0_ was the skinfold thickness of the saline injection at time 0.

On days 8 (basal concentration), 25 (primary response), and at 0 h on day 38 (secondary response), blood samples from vaccinated pigs were collected by orbital-sinus puncture into plasma vacutainer tubes containing an anticoagulant (EDTA, BD Vacutainer, BD, Franklin Lakes, NJ) for determination of plasma OVA-specific immunoglobulins G (IgG), OVA-specific IgG1, and plasma haptoglobin (Hp). Blood samples were stored on ice and then centrifuged for 20 min at 3,000 × *g* and 4 °C. Plasma was aliquoted into microcentrifuge tubes and stored at −20 °C until further analysis.

Plasma OVA-specific total IgG was determined using an indirect ELISA protocol as described by Begley et al. (2008). The high-affinity binding 96-well microtiter plates (Corning, Acton, MA) were coated with OVA at 500 μg/mL in carbonate buffer. All plasma samples were analyzed individually and diluted to 1/1,600 with phosphate buffered saline with polysorbate 20 (0.05% v/v; PBST) and conjugated IgG was diluted to 1/10,000 with tris(hydroxymethyl)aminomethane (TRIS) buffer. Plasma OVA-specific IgG1 was determined using an indirect ELISA protocol described by Lee et al. (2019). The high-affinity binding 96-well microtiter plates (Corning, Acton, MA) were coated with OVA at 10 μg/mL in carbonate buffer. All plasma samples were analyzed individually and diluted to 1/800 in PBST buffer, unconjugated IgG1 was diluted to 1/10,000 in PBST buffer, and alkaline phosphatase-conjugated IgG was diluted to 1/1,250 in TRIS buffer. For both sets of analyses, the reference sample was created by pooling plasma from all pigs on day 38. The basal samples were day 8 plasma samples, pooled within dietary treatment. The samples were tested in triplicate and the optical density was read at 405 nm using a Wallac 1420 Victor3 Multilabel counter (Perkin Elmer, Waltham, MA). The optical densities for individual samples were adjusted using a correction factor (Eq. 2). The average intra- and inter-assay variations were 2% and 5% for OVA-specific total IgG and 3% and 2% for OVA-specific IgG1, respectively.


$$\begin{array}{l} Correction\;factor=\frac{Overall\;mean\;of\;reference\;samples\;from\;all\;plates}{Actual\;mean\;of\;indivudal\;plate\;basal\;sample} \end{array}$$

Plasma Hp concentrations were determined by the clinical pathology services of the Animal Health Laboratory, University of Guelph, using a Roche Cobas c501 biochemistry analyzer (Roche Diagnostics, Indianapolis, IN).

### Diet Analysis

Diets were analyzed by SGS Agrifood Canada (Guelph, ON, Canada) for dry matter (AOAC, 2005; Method 930.15), crude protein (LECO-FP 428; LECO Instruments Ltd., Mississauga, ON, Canada; AOAC, 2005; Method 968.06), calcium, phosphorus, potassium, and magnesium (AOAC, 2005; Method 985.01). The samples were analyzed for mycotoxin content by Canadian Bio-Systems Inc. (Calgary, AB, Canada) using Agilent 1100 Series HPLC system (Agilent, Santa Clara, CA) and AB SCIEX 4000 MS/MS (SCIEX, Framingham, MA).

### Statistical Analysis

The GLIMMIX procedure of SAS (Version 9.4; SAS Inst. Inc., Cary, NC) was used for all statistical analyses. Data were analyzed among the LC diets using a 2 × 2 factorial with corn type (lDON vs. hDON), feed additive (− vs. +), and the interaction between corn type and feed additive as the main effects; unless stated otherwise, the interaction between corn type and feed additive was not significant for any outcome, therefore only the main effects of corn type and feed additive are presented. For Hp, OVA-specific IgG, and DHR, the model also included time and its interactions with corn type and feed additive; none of the time interactions were significant so only the main effect of time was presented. The random effect of block was included in all analyses, repeated measures were considered, and pen was used as the experimental unit. Pre-planned contrasts were also constructed to compare the main effects of corn type (lDON and hDON) and feed additive (− and +) to HC. A probability (*P*) of less than 0.05 was considered significant, whereas 0.05 ≤ *P* ≤ 0.10 was considered a tendency, and *P* > 0.10 was considered not significant.

## RESULTS

### Diets

The chemical analyses of nursery diets were generally comparable to calculated values and the Ca and P contents were within expected ranges allowing for analytical variation. With respect to DON contamination in the complete feeds, the HC diet in phases I and II contained less than 1 ppm of DON as intended; however, phase III contained 1.1 ppm DON, when the inclusion of corn was high (50%; Table 2). Despite aiming to achieve 1 ppm or less DON in the lDON diets, phases I and II were above 1 ppm and phase III contained between 0.7 and 0.8 ppm DON. The hDON diets in phases I, II and III averaged 3.4, 3.2, and 4.0 ppm DON, respectively, which were 11% greater in phase I and 25% less in phases II and III vs. the target DON contents.

**Table 2.** Analyzed nutrient composition and mycotoxin content of experimental diets (as-fed basis)^*a*^

Item	HC			lDON–			lDON+			hDON-			hDON+		
	PI	PII	PIII	PI	PII	PIII	PI	PII	PIII	PI	PII	PIII	PI	PII	PIII
Analyzed nutrient composition, %															
Dry matter	87.59	88.81	88.59	87.70	88.03	87.98	87.98	87.74	87.69	87.69	87.17	88.44	88.24	87.67	87.45
Crude protein	19.88	19.50	19.64	19.92	22.12	24.49	20.49	21.70	23.24	20.61	21.63	23.01	21.43	21.17	22.36
Calcium	0.82	0.74	0.61	1.00	0.94	0.73	1.02	0.85	0.83	1.02	0.87	0.84	1.04	0.85	0.82
Phosphorus	0.78	0.72	0.55	0.75	0.71	0.65	0.79	0.68	0.65	0.79	0.71	0.69	0.79	0.70	0.67
Potassium	0.86	0.76	0.76	0.88	0.93	1.00	0.91	0.95	0.95	0.90	0.95	1.00	0.91	0.93	0.97
Magnesium	0.13	0.14	0.16	0.17	0.19	0.18	0.17	0.19	0.19	0.17	0.19	0.18	0.17	0.19	0.18
Mycotoxin contents, ppm															
DON	0.5	0.5	1.1	1.5	2.4	0.8	1.3	1.7	0.7	3.8	3.2	4.7	3.0	3.2	3.2
Zearalenone	0.09	0.07	<0.05	0.16	0.12	0.09	0.06	0.10	0.10	0.24	0.15	0.31	0.09	0.23	0.29
Fumonisin	–	–	–	0.30	<0.20	<0.20	0.21	0.23	0.26	0.21	<0.20	<0.20	0.23	0.33	0.23
Aflatoxin B1	0.015	0.003	0.008	0.029	0.012	0.024	0.011	0.010	0.021	0.014	0.007	0.012	0.007	0.006	0.018

^*a*^Experimental diets: (1) high complexity (HC) diet containing multiple, highly digestible protein sources, (2) low complexity (LC) diets containing plant proteins as the main sources of protein, without (–) or with (+) the feed additive. Corn with high and low deoxynivalenol (DON) contents was used to create low and high contents of DON (lDON and hDON, respectively) in the LC diets to generate four diets according to a 2 × 2 factorial design with the feed additive and DON content as the factors. Phases (P) I, II, and III were fed between study days 0 and 7, 8 and 21, and 22 and 42, respectively.

### Growth Performance

There were no differences in initial BW among treatment groups and the feed additive did not affect growth performance in any phase of the nursery period. Pigs fed hDON diets had lower final BW than those fed lDON diets (*P* < 0.01) and tended to have lower final BW than pigs that received the HC diet (contrast; *P* = 0.052; Table 3). The ADG, ADFI, and G:F in phase I were not different for pigs fed hDON vs. lDON diets, but were less for pigs fed hDON and lDON diets vs. those fed the HC diet (contrasts; *P* < 0.05). In phase II, the ADG, ADFI, and G:F were not different for pigs fed hDON vs. lDON diets, but ADG was lower for pigs fed the hDON diet vs. those fed the HC diet (contrast; *P* < 0.05). Pigs fed the lDON diets had ADG not different from those fed the HC diet in phase II and there were no differences for ADFI or G:F for pigs fed either hDON or lDON diets vs. pigs fed the HC diet. In phase III and over the entire nursery period, ADG and ADFI were less for pigs fed hDON diets vs. pigs fed lDON diets (*P* < 0.05). In phase III and overall, ADG was less for pigs fed hDON diets vs. those fed the HC diet (contrast; *P* < 0.05), but the ADG of pigs fed lDON diets was not different from those fed the HC diet. In phase III, ADFI tended to be less for pigs fed hDON diets vs. those fed the HC diet (contrast; *P* = 0.099), but the ADFI of pigs fed lDON diets were not different from those fed the HC diet. Over the entire nursery period, ADFI was not different between pigs fed either hDON or lDON diets vs. those fed HC. In phase III and over the entire nursery period, G:F was not different for pigs fed hDON vs. lDON diets, and not different between pigs fed either hDON or lDON diets vs. those fed HC.

**Table 3.** Effect of nursery diets formulated with deoxynivalenol-contaminated corn and a feed additive on pig growth performance during the nursery period

Item	Dietary treatments^*a*^							
	HC	Corn type		Feed additive^*b*^		SEM^*d*^	*P-*value^*c*^	
		lDON	hDON	–	+		Corn	Feed additive
No.^*e*^	6	6	6	6	6			
Initial BW, kg	6.7	7.2	6.7	6.9	6.9	0.2	0.144	0.866
Final BW, kg	27.3	27.6	24.6^†^	26.2	26.0	0.5	0.001	0.812
ADG, g/d								
Phase I	99	18^*^	20^*^	16^*^	22^*^	12	0.883	0.715
Phase II	436	382	356^*^	371^*^	367^*^	24	0.179	0.814
Phase III	670	702	584^*^	640	646	23	<0.001	0.811
Overall	496	484	407^*^	440^*^	450^*^	19	0.010	0.711
ADFI, g/d								
Phase I	165	123^*^	119^*^	116^*^	126^*^	13	0.678	0.348
Phase II	565	530	507	521	516	14	0.581	0.928
Phase III	1094	1133	931^†^	1037	1027	60	0.028	0.905
Overall	738	769	651	708	712	34	0.022	0.935
G:F								
Phase I	0.58	0.10^*^	0.25^*^	0.21^*^	0.14^*^	0.12	0.384	0.684
Phase II	0.80	0.75	0.76	0.78	0.73	0.08	0.878	0.684
Phase III	0.58	0.63	0.65	0.63	0.65	0.03	0.739	0.674
Overall	0.64	0.63	0.63	0.63	0.63	0.02	0.848	0.858

^*a*^Experimental diets: (1) high complexity (HC) diet containing multiple, highly digestible protein sources, (2) low complexity (LC) diets containing plant proteins as the main sources of protein, without (–) or with (+) the feed additive. Corn with high and low deoxynivalenol (DON) contents was used to create low and high contents of DON (lDON and hDON, respectively) in the LC diets to generate four diets according to a 2 × 2 factorial design with the feed additive and DON content as the factors. Phases I, II, and III were fed between study days 0 and 7, 8 and 21, and 22 and 42, respectively.

^*b*^Contained a blend of vitamins, minerals, amino acids, antioxidants, organic acids, and absorbents; NutraMix (Canadian Bio-Systems Inc., Calgary, AB, Canada).

^*c*^Main effect *P*-values from 2 × 2 factorial analysis.

^*d*^Maximum standard error of the means.

^*e*^Number of experimental units (pens) for main effects.

^*^Differs from HC (contrast; *P* < 0.05).

^†^Tends to differ from HC (contrast; 0.05 ≤ *P* ≤ 0.10).

### Gut Morphology

Seven days after weaning, neither corn type (hDON vs. lDON) nor inclusion of the feed additive affected the relative organ weights or jejunal and ileal histomorphology (Table 4). There was however, an interactive effect of corn type and feed additive on the relative liver weight, where the additive reduced relative liver weights for pigs fed diets containing lDON corn but increased relative liver weights for pigs fed diets containing hDON corn (*P* = 0.042; data not shown). Pigs fed the lDON and hDON diets tended to have smaller relative full gut weights than pigs fed the HC diet (contrasts; *P* = 0.063 and *P* = 0.097, respectively), but relative empty gut weights were not different between pigs fed the lDON and hDON diets vs. those fed HC. The relative liver, stomach, and large intestine weights were not different between pigs fed the lDON and hDON diets vs. those fed HC, but pigs fed the lDON diets had lower relative small intestine weights compared to pigs fed the HC diet (contrast; *P* < 0.05). Jejunal villus heights were shorter for pigs fed hDON diets (contrast; *P* < 0.05) and tended to be shorter for pigs fed lDON diets (contrast; *P* = 0.090) compared to pigs fed HC. Crypt depth was not different between pigs fed the lDON and hDON diets vs. those fed HC, but the villus:crypt ratio tended to be less for pigs fed hDON diets vs. those fed HC (contrast; *P* = 0.091). Ileal villus heights were shorter for pigs fed hDON diets vs. those fed HC (contrast; *P* < 0.05) but were not different between pigs fed lDON diets vs. those fed HC. Ileal crypt depth and villus:crypt ratios were not different between pigs fed the lDON and hDON diets vs. those fed HC.

**Table 4.** Effect of nursery diets formulated with deoxynivalenol-contaminated corn and a feed additive on relative organ weights and jejunal and ileal morphology seven days after weaning

	Dietary treatments^*a*^							
	HC	Corn type		Feed additive^*b*^			*P-*value^*c*^	
Item		lDON	hDON	–	+	SEM^*d*^	Corn	Additive
Full gut, g/kg BW	164	147^†^	147^†^	149	145^†^	6	0.965	0.640
Empty gut, g/kg BW	71.5	68.1	68.4	68.4	68.0	1.9	0.890	0.884
Liver, g/kg BW^5^	24.7	23.9	23.7	23.9	23.7	0.5	0.784	0.847
Stomach, g/kg BW	7.66	6.84	7.60	7.53	6.91	0.38	0.177	0.259
Small intestine, g/kg BW	46.4	42.1^*^	44.0	42.5^*^	43.5	1.0	0.206	0.498
Large intestine, g/kg BW	17.3	16.9	16.9	17.3	16.6	0.5	0.952	0.316
Jejunal morphology, µm								
Villus height	538	466^†^	438^*^	435^*^	469^†^	17	0.304	0.198
Crypt depth	245	235	240	235	239	10	0.699	0.772
Villus:crypt ratio	2.22	2.03	1.87^†^	1.89	2.01	0.12	0.305	0.430
Ileal morphology, µm								
Villus height	502	453	420^*^	445^†^	428^*^	19	0.226	0.520
Crypt depth	245	238	226	229	235	12	0.446	0.684
Villus:crypt ratio	2.11	2.00	1.93	2.01	1.92	0.15	0.718	0.646

^*^Differs from HC (contrast; *P* < 0.05).

^†^Tends to differ from HC (contrast; 0.05 ≤ *P* ≤ 0.10).

^*a*^Experimental diets: (1) high complexity (HC) diet containing multiple, highly digestible protein sources, (2) low complexity (LC) diets containing plant proteins as the main sources of protein, without (–) or with (+) the feed additive. Corn with high and low deoxynivalenol (DON) contents was used to create low and high contents of DON (lDON and hDON, respectively) in the LC diets to generate four diets according to a 2 × 2 factorial design with the feed additive and DON content as the factors. Phases I, II, and III were fed between study days 0 and 7, 8 and 21, and 22 and 42, respectively. Each mean represents observations on 24 pigs (12 pigs for HC).

^*b*^Contained a blend of vitamins, minerals, amino acids, antioxidants, organic acids, and absorbents; NutraMix (Canadian Bio-Systems Inc., Calgary, AB, Canada).

^*c*^Main effect *P*-values from 2 × 2 factorial analysis.

^*d*^Maximum standard error of the means value.

^*e*^Liver, g/kg BW: lDON– 24.86, lDON+ 22.85, hDON– 22.89, hDON+ 24.43; additive decreased relative liver weights for pigs fed lDON diets but increased relative liver weights for pigs fed hDON diets (interaction; *P* = 0.042).

### Immune Response to OVA Vaccination

Among LC diets, plasma Hp was not affected by the main effects of corn type, the inclusion of the feed additive, or day of sample collection (Table 5) and no differences were observed between pigs fed the lDON and hDON diets vs. those fed HC at either day 8 or 25 after weaning. As expected, plasma OVA-specific total IgG concentrations were greater on day 38 (secondary response) than on day 25 (primary response; *P* < 0.001; Table 5). Among LC diets, the plasma OVA-specific total IgG was not affected by either the main effects of corn type or the inclusion of the feed additive. There were no differences in plasma OVA-specific total IgG concentrations between pigs fed the lDON and hDON diets vs. those fed HC on days 25 and 38 after weaning, but pigs fed diets without the immune-modulating feed additive had lower OVA-specific IgG compared to pigs fed HC, regardless of DON contents (contrast; *P* < 0.05). Among the LC diets, the plasma OVA-specific IgG 1 38 days post-weaning tended to have an interaction between corn type and the feed additive (*P* = 0.061; Table 5). Plasma OVA-specific IgG 1 tended to be less for pigs fed hDON compared to HC diets (contrast; *P* = 0.075). Among LC diets, the DHR to OVA was not affected by the main effects of corn type or feed additive but was influenced by the main effect of time (*P* < 0.001; Table 5). At any specific time point, there were no differences between pigs fed the lDON and hDON diets vs. those fed HC.

**Table 5.** Effect of nursery diets formulated with deoxynivalenol-contaminated corn and a feed additive on plasma haptoglobin concentration, plasma ovalbumin (OVA)-specific IgG and IgG1 response, and dermal hypersensitivity response to OVA post-weaning

Item	Dietary treatments^*a*^								
		Corn type		Feed additive^*b*^		SEM^*d*^	*P-*value^*c*^		
	HC	lDON	hDON	–	+		Corn	Additive	Time
No.^*e*^	6	6	6	6	6				
Haptoglobin, g/L							0.555	0.261	0.581
Day 8	0.67	0.68	0.67	0.74	0.61	0.11			
Day 25	0.76	0.80	0.65	0.81	0.65	0.12			
OVA-specific IgG^*f*^							0.991	0.194	<0.001
Day 25	0.10	0.10	0.11	0.12	0.09	0.07			
Day 38	0.92	0.84	0.83	0.74^*^	0.93	0.07			
OVA-specific IgG1^*g*^							0.186	0.694	–
Day 38	1.10	0.89	0.70^†^	0.82	0.77	0.18			
Change in SFT, mm^*h*^							0.844	0.337	<0.001
Hour 6	0.73	0.66	0.82	0.69	0.79	0.11			
Hour 24	0.61	0.47	0.45	0.40	0.52	0.10			
Hour 48	0.60	0.44	0.38	0.32	0.50	0.10			

^*a*^Experimental diets: (1) high complexity (HC) diet containing multiple, highly digestible protein sources, (2) low complexity (LC) diets containing plant proteins as the main sources of protein, without (–) or with (+) the feed additive. Corn with high and low deoxynivalenol (DON) contents was used to create low and high contents of DON (lDON and hDON, respectively) in the LC diets to generate four diets according to a 2 × 2 factorial design with the feed additive and DON content as the factors. Phases I, II, and III were fed between study days 0 and 7, 8 and 21, and 22 and 42, respectively. Each mean represents observations on 24 pigs (12 pigs for HC).

^*b*^Contained a blend of vitamins, minerals, amino acids, antioxidants, organic acids, and absorbents; NutraMix (Canadian Bio-Systems Inc., Calgary, AB, Canada).

^*c*^Main effect *P*-values from 2 × 2 factorial analysis.

^*d*^Maximum standard error of the means value.

^*e*^Number of experimental units (pens) for main effects.

^*f*^Corrected optical density.

^*g*^SFT = skin fold thickness; indicator of the dermal hypersensitivity response.

^*^Differs from HC (contrast; *P* < 0.05).

^†^Tends to differ from HC (contrast; 0.05 ≤ *P* ≤ 0.10).

## DISCUSSION

The objective of the current study was to evaluate the effects of simple, plant-based diets, DON contamination, and a feed additive containing immune-modulating components on growth performance, gut morphology, and indices of immune response in nursery pigs. Pigs fed low complexity (simple) diets (i.e. with SBM as the main protein source) with low DON contamination (i.e. ~1.5 ppm) experienced reduced growth performance (ADG, ADFI, and G:F) in phase I vs. those fed high complexity diets representative of typical commercial diets (i.e. with highly digestible sources of animal proteins). However, growth performance in phases II and III and over the entire nursery period was not different between pigs fed LC diets with low DON contamination and those fed the HC diet, such that, by the end of the nursery phase, the final body weights did not differ. Therefore, compensatory growth was evident for pigs fed the LC diets with low DON contamination. These results support the findings of Skinner et al. (2014) where pigs fed LC diets during the nursery period also compensated for the reduced growth early after weaning, though the compensation was not complete until the finishing stage. It is noted however, in the current study, the phase III HC diet contained DON contamination of 1.1 ppm, which is above the 1.0 ppm recommended threshold for growing pigs and twice the DON contents of the previous two phases. Therefore, it is possible the growth of the HC-fed pigs was compromised by DON in phase III, leading to sub-optimal ADFI and ADG, which allowed the pigs fed the lDON diets to catch up. Indeed, it appears that the initial exposure to DON has the greatest impact on feed intake followed by a gradual recovery when DON contamination was greater than 1 ppm in finishing pigs (Wellington et al., 2020). It is also noted however, that pigs fed the lDON diets in the current study still achieved growth performance (ADG, ADFI, and G:F) not different from pigs fed the HC diet in phase II when DON contamination was 2.1 (average) and 0.5 ppm for the lDON diets and HC diet, respectively. Conversely, pigs fed diets with high DON contents (i.e., between 3 and 5 ppm) were unable to achieve ADG or ADFI similar to those fed HC diets and had reduced BW at the end of the nursery period. Therefore, high DON contents in LC diets interfered with the ability to achieve compensatory growth when pigs received corn- and SBM-based nursery diets.

In the current study, all analyzed mycotoxins, other than DON, were below the recommended thresholds for nursery pigs, despite using naturally contaminated corn. Therefore, effects of dietary mycotoxin contamination on feed intake, relative organ weights, and indices of immune function can likely be attributed to DON. Indeed, pigs fed hDON diets (average DON contamination of 3.4, 3.2, and, 4.0 ppm in phases I, II, and III, respectively) had reduced ADG in all phases resulting in reduced body weights at the end of the nursery period vs. those fed the HC and lDON diets. Moreover, pigs fed the hDON diets had overall 13 and 18% lower ADFI vs. pigs fed HC and lDON diets, respectively. These reductions in ADG and ADFI are consistent with previous studies where nursery pigs were fed diets with >3.5 ppm of DON (Lessard et al., 2015; Van le Thanh et al., 2015). Since the overall G:F ratio was not different among pigs fed the experimental diets, the reduction in ADG was likely driven by reduced feed intake. The exception was during phase I where G:F was less for pigs fed both lDON and hDON diets vs. pigs fed HC. Though the measure of G:F is variable in phase I, this reduction in nutrient utilization efficiency corresponds to reduced jejunal villus heights for pigs fed hDON diets, and to a lesser extent, pigs fed lDON diets, after phase I (study day 7). Pigs fed the hDON diets also tended to have reduced villus height-to-crypt depth ratio. Together, these results indicate that some damage to the small intestinal morphology occurs when low complexity diets are fed to nursery pigs (likely due to the reduced feed intake and inflammatory response to the proteins in SBM; Li et al., 1991; Pluske et al., 1997; Lallès et al., 2004), but more extensive damage occurs when DON contamination is greater than 2.8 ppm, which is consistent with the findings of others (e.g., Bracarense et al., 2012). Since G:F was not different among dietary treatment groups after phase I, it is possible that the DON-induced damage to the small intestinal villi becomes less severe over time (e.g., Rotter et al., 1994; Kluess et al., 2016), though in the current study, tissues were not collected after phase I to confirm whether this was the case.

With respect to humoral immunity, OVA-specific IgG were detected in all pigs that were vaccinated with OVA, with an expected greater response observed on day 38 than on day 25. This indicated that the immunization protocol was efficacious; other researchers have also used a similar protocol in pigs (Crawley et al., 2005; Huber et al., 2018; Crosbie et al., 2021). Previous research demonstrated that DON contamination can compromise the immune responses in nursery pigs (Bondy and Pestka, 2000; Pestka et al., 2004; Cheng et al., 2006), but DON contamination can also have an immunostimulatory effect, depending on concentration and exposure duration (Bondy and Pestka, 2000). In the current study, there were no effects of low or high DON contamination on plasma concentrations of total OVA-specific IgG following immunization, which aligned with the results of others (Rotter et al., 1994; Gutzwiller et al., 2007; Zhang et al., 2020), though Lessard et al. (2015) demonstrated greater primary OVA-specific IgG responses for pigs fed 3.5 ppm DON with no effect on the secondary response. Conversely, in the current study, pigs fed hDON diets tended to have reduced plasma OVA-specific IgG1 on day 38 after weaning compared to pigs fed the HC diet. Since, protein antigens typically elicit an antibody-mediated response which primarily induce IgG1 (Vidarsson et al., 2014), assessing IgG1 in response to OVA (a protein antigen) is a more sensitive measure of humoral immunity vs. total IgG. Despite reducing plasma concentrations of OVA-specific IgG1, high DON contamination did not have a corresponding effect on the DHR response, which could be due to the increased variability of the measurement. Therefore, under the experimental conditions of the present study, it appeared that DON contamination between 3 and 5 ppm in LC nursery diets interfered, at least partially, with the humoral immune response toward a specific antigen.

The feed additive used in the current study was a blend of vitamins, yeast autolysate and an inorganic adsorbent designed to combat the ill effects of mycotoxicosis. For example, previous research has demonstrated that vitamin E partially reduced DON-induced oxidative DNA damage in immune cells (Frankič et al., 2008) and Van Le Thanh et al. (2015) demonstrated that a blend of amino acids, preservatives, and antioxidants improved growth performance of nursery pigs fed DON-contaminated diets. Additionally, certain yeast extracts have shown up to 19% DON binding capacity in vitro (Sabater-Vilar et al. 2007) and Jin et al. (2017) found improved growth performance of pigs fed a blend of organic acids and inorganic and organic absorbents when fed a DON-contaminated diet. In the current study, the feed additive had minimal effects on growth performance or intestinal morphology and consequently, did not rescue the growth performance of pigs fed LC diets with low (phase I only) or high DON contamination vs. those fed high complexity diets. Conversely, the absence of the feed additive in LC diets resulted in 1.2× lower plasma OVA-specific IgG, which may indicate a less robust humoral immune response to vaccination when the feed additive was not included in LC diets, regardless of DON contamination level.

Finally, pigs in the current study were from a high-health herd, and the low complexity diets and DON contamination were not sufficient to alter the plasma Hp concentrations. Haptoglobin is an acute-phase protein whose concentration increases during the innate acute-phase response (Piñeiro et al., 2009). In the current study, the plasma Hp concentrations were either lower, or within the reference range recommended by Piñeiro et al. (2009), indicating that despite the DON contamination, these pigs were not as challenged as those from commercial herds. Similarly to this study, Wu et al. (2015) found no difference in Hp concentrations of growing pigs when DON contamination was 3 ppm, but greater Hp concentration when DON contamination was above 6 ppm. Therefore, it is possible that the addition of an immune challenge or, other stressors such as overcrowding and transportation etc., for pigs fed LC diets contaminated with low or high levels of DON could interfere with the ability to achieve compensatory growth and cause further detriment to gut morphology; under such a scenario, the feed additive could possibly provide a more obvious benefit.

## CONCLUSION

In conclusion, nursery pigs fed low complexity, corn-and-SBM-based nursery diets with DON contamination around 1.5 ppm were able to exhibit compensatory growth and achieve nursery exit body weights not different from pigs fed high complexity nursery diets that included highly digestible protein sources. Therefore, it is possible that nursery diets can be simplified, even when corn is contaminated with DON, as long as the complete diets do not exceed ~1.5 ppm DON. Such low complexity diets could be used as a means to reduce nursery feed costs. However, when DON contamination exceeds 3 ppm in low complexity nursery diets, compensatory growth was not achievable within the nursery period. Moreover, at these DON levels and in low complexity diets, the feed additive was unsuccessful at rescuing growth performance or small intestinal morphology.
